# Adhesion of Immature and Mature T Cells Induces in Human Thymic Epithelial Cells (TEC) Activation of IL-6 Gene Trascription Factors (NF-κB And NF-IL6) and IL-6 Gene Expression : Role of αtβ1 and α6β4 Integrins

**DOI:** 10.1155/2000/48239

**Published:** 2000

**Authors:** Emma Fiorini, Pier Carlo Marchisio, Maria Teresa Scupoli, Ornella Poffe, Elda Tagliabue, Monica Brentegani, Marco Colombatti, Franscesco Santini, Giuseppe Tridente, Dunia Ramarli

**Affiliations:** 1 Section of Immunology Dept of Pathology University of Verona Verona 37134 Italy; 2 DIBIT Department of Biological and Technological Research San Raffaele Scientific Institute Milano 20132 Italy; 3 Clinical Immunology Azienda Ospedaliera Verona Verona 37134 Italy; 4 Oncologia Sperimentale E-Istituto Nazionale Tumori Milano Italy; 5 Cardiosurgery Division University of Velona Italy; 6 Servizion di Immunologia Clinica Policlinico G.B. Rossi Via delle Menegone Verona 37134 Italy

## Abstract

T cell precursors homed to thymus develop in close contact with stromal cells. Among them, thymic epithelial cells (TEC) are known to exert dominant roles in their survival and functional shaping. Key molecules mediating TEC/thymocytes interactions include cytokines and growth factors secreted by the two cell types and adhesion receptors mediating cell contact. Signaling events triggered in thymocytes by adhesion to epithelial cells have been extensively investigated, whereas little is known on the opposite phenomenon. We have previously investigated this issue in a co-culture system composed of TEC cultures derived from human normal thymus and heterologous thymocytes. We demonstrated that thymocytes adhere to TEC involving β1 and β4 integrins and induce the clustering of (α3β1 and α6β4 heterodimers at the TEC surface. In addition thymocyte adhesion was followed by activation of NF-κB and NF-IL6 gene transciption factors and enhanced IL-6 production. The two latter phenomena were reproduced by the cross-linking of the α3, α6, β1 and β4 integrins, thus implying that the α3β1 and α6β4  heterodimers can signal during thymocyte adhesion. We have extended our previous work investigating in the same experimental setting the inducing activity of non stimulated or activated policlonal or clonal mature T cells as representative of the more mature thymocyte subset. We found that adhesion of unstimulated T cell i) involved β1, but not β4 integrin functions at the surface ii) induced the clustering of α3β1 , but not α2β1  heterodimers at the TEC surface and iii) up-regulated the nuclear binding activity of NF-κB transcription factor and the IL-6 secretion. We propose that α3β1  and α6β4  heterodimers are induced to cluster at the TEC surface recognizing yet unknown cellular ligands differentially expressed during T cell development.


*Developmental Immunology*, 2000, Vol. 7(2-4), pp. 195-208
Reprints available directly from the publisher
Photocopying permitted by license only

© 2000 OPA (Overseas Publishers Association)
N.V. Published by license under the
Harwood Academic Publishers imprint,
part of the Gordon and Breach Publishing Group.
Printed in Malaysia

### Adhesion of Immature and Mature T Cells Induces in Human Thymic Epithelial Cells (TEC) Activation of IL-6 Gene Trascription Factors (NF-κB And NF-IL6) and IL-6 Gene Expression : Role of α3β1 and α6β4 Integrins

EMMA FIORINI[a], PIER CARLO MARCHISIO[b], MARIA TERESA SCUPOLI[a], ORNELLA POFFE[a], ELDA TAGLIABUE[d], MONICA BRENTEGANI[c], MARCO COLOMBATTI[a], FRANSCESCO SANTINI[a], GIUSEPPE TRIDENTE[a] and DUNIA RAMARLI[c]*

[a]*Section of Immunology, Dept of Pathology, University of Verona, 37134 Verona, Italy,* [b]*DIBIT, Department of Biological and Technological Research, San Raffaele Scientific Institute, 20132 Milano, Italy,* [c]*Clinical Immunology, Azienda Ospedaliera Verona, 37134 Verona, Italy,* [d]*Oncologia Sperimentale E-Istituto Nazionale Tumori, Milano, Italy and* [e]*Cardiosurgery Division University of Velona*

T cell precursors homed to thymus develop in close contact with stromal cells. Among them, thymic epithelial cells (TEC) are known to exert dominant roles in their survival and functional shaping. Key molecules mediating TEC/ thymocytes interactions include cytokines and growth factors secreted by the two cell types and adhesion receptors mediating cell contact. Signaling events triggered in thymocytes by adhesion to epithelial cells have been extensively investigated, whereas little is known on the opposite phenomenon. We have previously investigated this issue in a co-culture system composed of TEC cultures derived from human normal thymus and heterologous thymocytes. We demonstrated that thymocytes adhere to TEC involving β1 and β4 integrins and induce the clustering of α3β1 and α6β4 heterodimers at the TEC surface. In addition thymocyte adhesion was followed by activation of NF-κB and NF-IL6 gene transciption factors and enhanced IL-6 production. The two latter phenomena were reproduced by the cross-linking of the α3, α6, β1 and β4 integrins, thus implying that the α3β1 and α6β4 heterodimers can signal during thymocyte adhesion. We have extended our previous work investigating in the same experimental setting the inducing activity of non stimulated or activated policlonal or clonal mature T cells as representative of the more mature thymocyte subset. We found that adhesion of unstimulated T cell i) involved β1, but not β4 integrin functions at the surface ii) induced the clustering of α3β1, but not α2β1 heterodimers at the TEC surface and iii) up-regulated the nuclear binding activity of NF-κB transcription factor and the IL-6 secretion. We propose that α3β1 and α6β4 heterodimers are induced to cluster at the TEC surface recognizing yet unknown cellular ligands differentially expressed during T cell development.

### INTRODUCTION

The molecular events that mediate the homing, the development and the functional shaping of human T cell precursors have been extensively investigated in the last few years, showing the instrumental role exerted in these processes by the stromal components of the thymus, specially by the thymic epithelial cells

* Corresponding author: Dr. Dunia Ramarli, Servizion di Immunologia Clinica, Policlinico G.B. Rossi, Via delle Menegone, 37134 Verona. Tel 39–45 8074007, FAX 39–45 590900, e-mail dunia@borgoroma.univr.it

(TEC) (Boyd et al, 1993). More recently, it has became evident that a two-way interaction occurr between TEC and thymocytes, in such a way that thymocytes themselves act as a cellular constraints required for proper development of thymic epithelium. TEC differentiation and T-cell commitment appear to be interdependent during the thymic organogenesis. Studies in SCID, human CD3ε-transgenic or RAG (-/-) knockout mice have shown in fact that the blockage of thymocyte differentiation at the DN $CD44^{+}$ $CD25^{-}$ stage determines the lack of organization of the cortical epithelium whereas that at the $CD44^{-}CD25^{+}$ stage results in the absence of medullary epithelium (Hollander et al, 1995, Penit et al, 1996, Klug et al, 1998). Thymic epithelium is composed of different subsets, interconnected by desmosomes and surrounded by extracellular matrix, forming an intralobular network filled with developing thymocytes (Boyd et al, 1993). The molecular interactions between thymocytes and the subcapsular, cortical or medullary TEC are far from being elucidated because the pathways of human TEC differentiation are still uncertain and the TEC progenitors remain unidentifyed. However, key elements that might be involved in all these interactions are the cytokines, growth factors and neuropeptides differentially secreted by the two cell lineages ( Savino et al, 1998, Hadden J.W., 1998) and the multiple adhesion receptors (i.e. member of the Ig superfamily, integrins and cadherins) interacting at the single cell level ( Patel D.D. and Haynes B.F., 1993, Lee et al, 1994, Salomon et al, 1997).

TEC produce multiple cytokines and growth factors and express a wide number of cytokine, or adhesion receptors. Several cytokines and growth factors (i.e. IL-1β, IL-6, EGF, NGF) have been investigated as regard their activity in promoting TEC expansion, differentiation or expression of endogenous cytokine genes (Screpanti et al, 1992 and 1995, Cohen-Kaminsly et al, 1993 ). Less is known regarding the activity of adhesion receptors.

Previous reports by us and others indicated that TEC derived from normal or myasthenic thymus increase the phosphorylation of cytoplasmic proteins or the production of IL-6 following the coculture with thymocytes or neoplastic T cells (Couture et al, 1992, Cohen-Kaminsky et al 1993, Ramarli et al, 1996). Based on these observations we put forward the hypothesis that adhesion receptors at the TEC membrane initiate these phenomena. Among them, we focussed on integrins as trigger-elements at the membrane level, and on IL-6 as a target gene, in consideration of: i) the known ability of integrins to induce cytokine gene expression in other adherent cells (Defilippi et al, 1997), and ii) the importance of IL-6 within the thymic microenvironment, due to its activities on activation, survival and cytotoxic differentiation of lymphoid and epithelial cells (Henttinen et al 1995, Adkins et al 1996, Akira, S and Kishimoto T, 1997). The IL-6 gene expression is regulated at a transcriptional level by the cooperative activity of NF-κB and NF-IL6 transcription factors (Akira, S and Kishimoto T, 1997). Both transcription factors undergo kinase-dependent, post-transcriptional modification to function. Tyrosine or serine phosphorylation of inhibitors of NF-κB (IκBs) is needed to disrupt the cytosolic IκB-NF-kB complexes thereby allowing p50 /p65 active NF-κB heterodimers to enter the nucleus (Baldwin AS, 1996). Extensive serine/threonine phosphorylation is required to regulate the transactivation potential and the DNA binding activity of the four NF-IL6 isoforms so far decribed in epithelial and fibrosarcoma cell lines (Akira, S and Kishimoto T, 1997). Adhesion-dependent induction of IL-6 gene expression was investigated in normal TEC at trascriptional and protein levels, analysing the time-dependent activation of IL-6 gene transcription factors and the protein secretion. We found that heterologous thymocytes : i) adhere to TEC involving β1 and, to a lesser extent, β4 integrins at the membrane level; ii) induce the clustering of α3β1 and α6β4 heterodimers at the TEC surface. We also found that adhesion of thymocytes, but not their soluble factors, induced the activation of IL-6 gene transcription factors and the IL-6 gene expression (Ramarli et al, 1998). The cross-linkings of α (α3,α6) or β (β1,β4) integrins mediated by mAbs reproduced the two latter phenomenon. These observations implyed that TEC/thymocytes mutual interactions may regulate the availability of IL-6 within the microenvironment *via*

TEC induction triggered by β1 and β4 clustering at the cell surface. The thymocyte ligand/s recognized by TEC integrins remain unidentifyed. However, to investigate whether the/se ligand/s were expressed in a stage-, activation- or microenvironment-dependent manner we extended our previous studies, analysing in the same experimental framework the inducing activity of circulating mature T cells, freshly isolated or driven to proliferation by mitogen treatment or antigen recognition. T cells were chosen, although they represent a mature thymocyte subset only, in order to avoid the use of harsh fractionation procedures which might alter the membrane reactivity and/or the apoptotic rate of thymocyte subpopulations. We found that TEC grown in organized, integrin-polarized monolayers bind in a time dependent manner and with increasing rate to unfractionated thymocytes, non stimulated T cells, policlonally stimulated T cells or Ag-stimulated clonal T cells. The TEC binding to the various T cells i) involved β1 integrin function at the surface, whereas the b4 contribution was irrelevant ii) induced the clustering of α3β1, but not α2β1 heterodimers at the TEC surface and iii) was associated with increased nuclear binding activity of NF-κB transcription factor, known to be required for maximal expression of IL-6 gene expression in inducible systems (Akira, S and Kishimoto T, 1997). Based on previous and present results we propose that i) α3β1 integrins expressed at the TEC surface recognize cellular ligand/s that are present on thymocyte populations most likely including the phenotypically mature subset and maintained by circulating normal T cells whereas ii) α6β4 integrins recognize on thymocytes ligand/s restricted to stage- or microenvironment-dependent expression.

### MATERIALS AND METHODS

### Cell isolation and cultures

Thymic epithelial cell cultures were derived from patients (age <5 yr) undergoing corrective cardiosurgery as previously described (Green et al, 1979). Briefly, tissue specimens were minced and trypsinised (0.05% trypsin/0.01% EDTA) at 37°C for 3 h. Cells were collected every 30 min, pooled, plated onto lethally irradiated 3T3-J2 cells (gift of Prof. H. Green, Harvard Medical School, Boston, MA) at $2.5x10^4/cm^2$ and cultured in humidified atmosphere of 5% $CO_2$ in growth medium composed of DMEM/Ham's F12 media (3:1 mixture), 10% FCS, insulin (5 μg/ml), transferrin (5 μg/ml), adenine (0.18 μM), hydrocortisone (0.4 μg/ml), cholera toxin (0.1 nM), triiodothyronine (2 nM), Epidermal Growth Factor (10 ng/ml), glutamine (4 mM), and antibiotics. From the $3^{rd}$ passage cells were plated in the absence of feeder-layer cells and grown in one thirds of insulin, transferrin, adenine, hydrocortison, cholera toxin, triiodothyronine and EGF. These cells were used for experimental assays. Media were purchased from Seromed (Berlin, FRG) and supplements from SIGMA-Aldrich (Milano, Italy). EGF was from Austral Biological (San Ramon, CA). Human thymocytes were prepared by mechanical disruption of fresh thymus specimens. At least 95% viable cells were isolated from the cell suspension by Ficoll-Hypaque gradient, washed and used immediately after preparation. T cells were isolated from normal peripheral blood mononuclear cells (Pbl) by E-rosetting and one-step Ficoll-Hypaque gradient. Purified T cells were used whithin 24 h from isolation (also referred as T cells in the text) or after stimulation with PHA (5μg/ml, Murex, Pomezia, Italy) and rIL-2 (200 U/ml) ( generously provided by Chiron, Milano, Italy) for 7–10 days. MBP (Myelin basic protein) specific T cell clones were isolated by plating in limiting dilution (0.3 cell/96 wells Costar plate) Pbl (obtained from a patient with Multiple Sclerosis) previously stimulated for a week with 100 μgs/ml of MBP( Sigma) in RPMI-1640 at 10% human AB serum and a second week with rIL-2 (100 IU/ml). Wells positive for cell growth were further expanded, analysed for specific Ag recognition and used as cloned T cells. Clonality was assessed by statistical methods. T cells, PHA-IL-2 activated and Ag-activated T cells were analysed by cytofluorometry for the expression of MHC-class II, CD3, CD4, CD8, ICAM-1, β2 chain of LFA-1 complex and β1 integrins.

### Chemicals and Antibodies

The following antibodies were used for immunostaining, cell treatment, Western blotting, Electromobility Shift Assay : mAbs anti CD2, CD3, CD4, CD8, CD16 and FITC-labelled F(ab)2 goat anti mouse Ig from Becton-Dickinson (Mountain View, CA); anti CD1a, CD18, ICAM-1, VCAM-1 and anti α3 (CD49c) and anti α2 (Gi9) from Immunotech Int. (Marseille, France); mAbs B9–12 (anti MCH- Class I) (Lemonnier et al, 1982) and D1–12 (anti MHC Class II), provided by Dr. R.S. Accolla, University of Pavia at Varese, Italy; mAb 3E1 (anti β4) from Calbiochem ( Calbiochem, La Jolla, CA); mAb MAR 4 (anti β1) and MAR 6 (anti α6), kindly provided by Dr. S. Ménard, Istituto Nazionale Tumori, Milano, Italy; mAb Lam89 (anti laminin 1) from Sigma; mAb GB3 (anti laminin 5), gift of Dr. Patrick Verrando, University of Nice, France; $F(ab)_2$ goat anti mouse Ig from Pierce (Pierce, Oud Beijerland, The Netherlands); swine anti rabbit or rabbit anti-mouse IgG for immunoistochemistry from Dako, Glostrup, Denmark; rabbit antiserum anti-cingulin, gift of Dr. S. Citi, University of Padova, Italy; antisera anti NF-IL6 (C19) and anti NF-kB p65 (A) and p50 from Santa Cruz Biotechnology, Santa Cruz, CA; mAb anti phosphoserine in agarose-coupled or uncoupled form, horseradish peroxidase-labelled rabbit anti mouse, goat anti-rabbit antisera and non specific mouse Immunoglobulins from SIGMA. Flow cytometry was performed in a FacScan flow cytometer (Becton-Dickinson).

### Binding Assays

Epithelial cells were plated into 24-wells plates (Costar, Cambridge, MA) at high density ($5x10^5$ cells/well) 24 h before assays. T cells were labelled overnight with [$^{14}$C]-leucin (NEN™, Life Products, Boston MA) (310.86 mCi/mMole, 10mCurie/$10^6$cells) in RPMI containing 10% FCS and 10 mM Hepes. Labeled cells were washed and resuspended at $10^5$ cells/ml in RPMI, 10 mM Hepes and 3% BSA. Labelled cells were dispensed on each well at the indicated TEC/T cell ratio and allowed to adhere for the indicated times at 37°C. Nonadherent cells were removed by gentle pipetting (sample A) followed by three washes with binding medium (sample B). Adherent cells were solubilized by addition of 100ml of 1% SDS (sample C). Non adherent cells (A), washes (B) or adherent cells (C) were mixed with Filter Count CSC cocktail (Packard Instrument, Meriden, CT) and counted in a liquid scintillation counter Wallac 1409 (Wallac, Turku, Finland). The percentage of lymphoid cell binding was calculated as cpm of sample C/cpm of sample (A + B + C).

### Immunostaining of TEC/T cell cocultures

Cocultures carried on overnight at 37°C humidified atmosphere of 5% CO2 at 1:1 TEC:T cell ratio, were fixed in 3% paraformaldheyde (electron microscope grade), 2% sucrose in PBS pH 7.6 for 5 min at room temperature and permeabilized (3 min, 4°C) in Hepes-Triton X-100 buffer (20 mM Hepes, 300 mM sucrose, 50 mM NaCl, 3 mM MgCl2, 0.5% Triton X-100, pH 7.4). Staining for F-actin was performed with fluorescein-labeled phalloidin (F-PHD; Sigma) (200 nM for 20 min at 37°C in the dark). Adhesion molecules were detected with the relevant mAbs (see above) followed by rhodamine-tagged swine anti rabbit or rabbit anti-mouse IgG. Primary antibodies were replaced by mouse IgG or preimmune rabbit sera in control samples. Coverslips were incubated with the appropriate rhodamine-tagged secondary antibodies routinely supplemented with 200 nM F-PHD. Afterwashing, coverslips were mounted in Mowiol 4–88 (Hoechst, Frankfurt am Main, Germany) and observed in a Zeiss Axiophot microscope equipped for epifluorescence and a 63x planapochromatic lens. Stained coverslips were photographed with Kodak T-MAX 400 films exposed at 1000 ISO and developed at 1600 ISO in T-MAX developer for 10 min at 20°C. The same coverslips were analyzed in parallel with a confocal laser scanning microscope (CLSM Bio-Rad 1024). Image files were recorded on different channels, digitally reconstructed to provide z-axis views and printed with ADOBE Photoshop 3.5.

### Cell extracts and Electrophoretic Mobility Shift Assay (EMSA)

TEC monolayers were cocultured with the various T cells for the indicated times. After removing T cells by extensive vigorous washings, cell extracts were prepared as previously described ( Schreiber et al, 1989) from TEC detached by trysin-EDTA treatment and scored negative for $CD2^+$ contaminants by cytofluorometry. Protein concentration was assessed by Coomassie protein assay reagent (Pierce, Rockford, IL). DNA binding activity was determined by using a $^{32}P\gamma$-ATP end-labelled double stranded oligoprobe containing the κb site of the IL-6 promoter. Six μgs of cell extracts were incubated for 30 min at room temperature with the probe ($1x10^5$ cpms/sample) in 20 μl of binding buffer (20 mM Tris-HCl pH 7.5, 0.1 M NaCl, 1 mM DTT, 1 mM EDTA, 1 mg/ml BSA, 0.1% NP-40, 4% glycerol) containing 1 μg of Poly (dI-dC) (Pharmacia, Uppsala, Sweeden). Nucleoprotein complexes were electrophoresed on a 5% polyacrylamide (30:1.2) gel in 0.05 M Trisborate/1 mM EDTA at 150 V. Gels were dried and exposed to Amersham Hyperfilm films (Amersham, Little Chalfont, England). Film densitometry was performed with an UltroScan Densitometer and the built-in software (LKB, Bromma, Sweden).

### Cell labelling, Immunoprecipitation and Western blotting

TEC monolayers were labelled overnight with 25 μC/ml of $^{14}C$-leucine (324.9 mC/mMole) (NEN Research Dupont, Boston, MA) in medium. Cell lysates were prepared as for EMSA. Twenty μgs of nuclear extracts were pre-cleared with 20 μl of swollen Protein A-Sepharose beads (Pharmacia) in lysis buffer (20 mM Tris-HCl pH 7.2, 0.5 mM DTT, 1 mM EDTA, 0.5 mM PMSF adjusted to 0.15 M NaCl) for 1 h at room temperature and incubated with the anti NF-IL6 antiserum at 1: $10^3$ dilution for additional 2 h on ice. Immunocomplexes were precipitated with 20 μl of Protein A-Sepharose beads for 1h at room temperature and extensively washed in 0.05 M Tris-HCl pH 7.2, 0.15 M NaCl, 0.1% NP40. Immunoprecipitates (at equal amount of counts) were fractionated by 12%-SDS-PAGE, electroblotted to nitrocellulose membrane (BioRad, Milano, Italy) and probed with anti phosphoserine mAbs peroxidase-labelled goat anti mouse-Igs in TBST 1.5% w/v powdered low fat dry milk. Membranes were washed with TBST buffer and specific bands revealed by Enhanced Chemiluminescence system (ECL, Amersham).

### IL-6 production

Cocultures destined to IL-6 production assays were carried on 12 h. After T cell removal, TEC were recovered by trypsin-EDTA, analyzed for the absence of contaminants as above and plated onto new plates. Cell supernatants were collected 24 and 72 h from re-plating and assayed for IL-6 production by ELISA kit according to manufacturer's instructions (CLB, Amsterdam, The Netherlands). OD values were plotted on the standard curve and expressed as $ng/10^6$ cells recovered.

### RESULTS

Circulating mature T cells were compared to immature thymocytes for the ability 1) to reproduce the inducing activity on IL-6 gene expression in TEC and 2) for the recruitment of $\alpha3\beta1$ and $\alpha6\beta4$ integrin heterodimers at the TEC membrane.

We firstly assessed whether differences in the T cell activation influenced the lympho-epithelial adhesion. To this purpose, binding assays were performed between TEC grown to tightly confluent monolayers and i) unfractionated freshly isolated thymocytes, ii) non stimulated purified T cells, iii) PHA-IL2-activated T cells or Ag-specific activated clonal T cells. Adhesion partners were preliminary analysed by cytofluorometry for the expression of lineage-specific or activation antigens and adhesion molecules (not shown). Freshly isolated, unfractionated thymocytes comprised 21±7 (SD) % of CD3 negative, 53±4% of $CD3^{low-intermediate}$ and 25±8.5% $CD3^{high}$ positive cells, all expressing the β2 chain of LFA-1 complex and low level of β1 integrins. Purified T cells (>94%

CD3+) uniformly expressed high amounts of β2 chain of LFA-1 but very low levels of ICAM-1 and β1 integrins. Activated T cells and Ag-specific T cell clones (the latter 100% CD4 positive) shared the coexpression of high amounts of MHC-class II antigens with β2 integrins (>80% and >95% respectively) as well as the expression of β1 integrins (53±33 SD and 60±39 SD, respectively). The extent of ICAM-1 expression was higher on mitogen than in Ag activated clonal T cells (71±28 SD % vs 46±32 SD%). TEC uniformly expressed high amounts of MHC-class I, β1 and α3 integrins with moderate amounts of α5. Seventy-80% of the cells expressed α6 and β4 integrins whereas 9.5–20% were found to be positive for ICAM-1. Virtually all cells lacked CD18, CD16, VCAM and MHC-class II antigens.

### TEC/THYMOCYTE AND TEC/T CELL ADHESION

As shown in Table I, lympho-epithelial adhesion occurred quickly with all T cells examined, progressively increasing from the lowest levels observed at 30 min with thymocytes or T cells (18±1.5SE % and 33±2.5SE % respectively) to the intermediate levels of mitogen-activated policlonal T cells (39±2.5SE %) up to the highest levels found in Ag-activated clonal T cells (46±5.3SE%). All lympho-epithelial adhesions increased after 1h, yet maintaining a difference between the level of thymocytes (38±2.4) and the similar plateau reached by non stimulated and activated T cells ( range : 45±3.1–50±0.8% ). As we have previously demonstrated that β1 and β4 integrins belong to the group of molecules mediating TEC adhesion to thymocytes, (Ramarli et al, 1998) we next investigated whether the same molecules were involved in the TEC adhesion to mature T cells.

### TEC/T CELL ADHESION INVOLVES INTEGRIN OF THE β1 FAMILY

The function of β1 and β4 heterodimers in TEC-T cell binding could not be directly investigated (i.e. by blocking experiments with inhibiting peptides), because they both recognize conformational structures (Branderberger et al 1996, Delwel G.O.) and it was hence evaluated by mAbs recognizing their extracellular domains. Non specific mouse Igs and anti ICAM-1 mAbs were respectively used as additional negative and positive controls. TEC monolayers were used precoated with the different mAbs for 30 min at 4° C. Values of non specific mouse Igs were subtracted from those of mAb-treated samples. As shown in Table II, TEC/T cell bindings were all inhibited by anti β1 mAbs, but to a very different extents. Great inhibitions were observed with thymocytes or non stimulated T cells (43±1SE% and 36±7SE% respectively) whereas low inhibitions were observed with mitogen (12±3.4 SE%) or Ag-activated T cells (9±0.1SE%). Anti β4 mAbs inhibited TEC/thymocytes binding (26±1.5%), but failed in the case of T cells or Ag-specific clones or showed very low activity in the case of activated T cells (9±2%). MAbs anti ICAM-1 consistently inhibited TEC interaction with thymocytes, activated or Ag-activated T cells, whereas lacked activity in the case of non stimulated T cells. These results indicated that integrins of the β1 family were still recruited in the TEC/T relationship with T cells, whereas α6β4 heterodimers seemed to be more restricted in that with thymocytes. Based on the results obtained in binding and binding inhibition studies we selected the unstimulated T cells for further investigations. First, it was assessed whether their adhesion may trigger β1 repolarizations at the cell contact sites.

TABLE I TEC binding to unfractionated thymocytes, non stimulated T cells, Tcells activated by PHA IL2 treatment or T cell clones activated by antigen (MBP) recognition

| *Cells* | *TEC: lymphoid cell ratio* | *% of binding* | |
|---|---|---|---|
| | | 30 min | 1h |
| TEC/thymocytes | 1:5 | 18±1.5[a] | 38±2.4[a] |
| TEC/T cells | 1:1 | 33±2.5 | 48±3 |
| TEC/activated T cells | 1:1 | 39±2.5 | 45±3.1 |
| TEC/MBP-T cell clones | 1:1 | 46±5.3 | 50±0.8 |

a. Values represent the mean± SE obtained from three indepent experiments performed in duplicate with TEC and T cells obtained from independent donors. Ag-specific T cell clones include three different clones obtained from a single donor.

TABLE II Inhibition of TEC/T cell binding by anti β1. β4 or ICAM-1 mAbs

| *Cells* | *TEC:lymphoid cell ratio* | *% of binding*[a] | *% of binding decrease*[b] |
|---|---|---|---|
| TEC/thymocytes | 1:5 | 38±2.4 | |
| + anti β1 mAb | 1:5 | 16±1.5 | 43±1 |
| + anti β4 mAb | 1:5 | 23±4 | 26±1.5 |
| + anti ICAM-1 | 1:5 | 23±7 | 24.5±3 |
| TEC/T cells | 1:1 | 48±3 | |
| + anti β1 mAb | 1:1 | 27±8 | 36±7 |
| + anti β4 mAb | 1:1 | 44±0.8 | 0.3±0.1 |
| + anti ICAM-1 | 1:1 | 43±1.5 | 3.3±1 |
| TEC/activated T cells | 1:1 | 45±3.1 | |
| + anti β1 mAb | 1:1 | 35±4 | 12±3.4 |
| + anti β4 mAb | 1:1 | 27±6.1 | 9±2 |
| + anti ICAM-1 | 1:1 | 31±2 | 32±5 |
| TEC/MBP-T cell clones | 1:1 | 50±0.8 | |
| + anti β1 mAb | 1:1 | 42±1.1 | 9±0.1 |
| + anti β4 mAb | 1:1 | 48.1±1 | 0 |
| + anti ICAM-1 | 1:1 | 32±4.4 | 32±4.2 |

a. Mean values±SE of three experiments performed with TEC and T cells obtained from different donors. Values of Ag-specific T cells were from three clones derived from a single patient with Multiple Sclerosis.

b. The percentage of binding decrease is calculated finally subtracting the not specific decrease observed in the presence of unrelated, total mouse Ig. Values were 15±2, 8±3, 9±4 and 5.5±1.9 respectively for thymocytes, T cells, activated T cells and Ag-specific T cells.

### T CELL ADHESION INDUCES α3β1, BUT NOT α2β1 INTEGRIN CLUSTERING AT THE CELL BOUNDARIES

TEC /Tcells cocultures were immunostained with mAbs recognizing α (α2, α3, α4) and β1 integrins and examined by optical microscopy (Figs 2). Previous immunohystochemical studies of TEC monolayers have shown that α3β1 and α2β1 lined the TEC intercellular boundaries codistributing with the microfilamentous network (Ramarli et al, 1998). Adjoining thymocytes selectively remodeled this pattern, in a way that α3β, but not α2β1, clustered at the TEC/thymocyte contact sites, still maintaining its lateral distribution (ibid). The same phenomenon was observed in the case of T cells. As shown in panels b only T cells expressed α4β1. α3β1, but not α2β1 (not shown) lined both the intercellular TEC boundaries and the interface between TEC and T cells (panels d and f), codistributing with the microfilamentous network (see F-actin staining in panels e and f). This finding prompted the search for laminin 1 and 5, fibronectin and Collagen IV in between TEC and T cells. All proteins were found located at TEC attachment surface while neither was found interposed at the TEC-T cell interface (not shown), thus indicating that α3β1, among the considered surface antigens, was selectectively induced to cluster at the TEC-T cell contact sites by an unknown mechanism that apparently excluded any known bridging component of the extracellular matrix. Based on these findings the T cell adhesion was compared with that of thymocytes as the inducing activity on IL-6 gene expression.

Fig. 2 summarizes previous results concerning the activation of IL-6 trascription factors (DNA nuclear binding activity of NF-κB and phosphorylation of NF-IL6 43 KDa isoform) and the enhancement of IL-6 secretion observed following i) adhesion of thymocytes or ii) α (α3, α6) or β (β1, β4) ligation or cross-linking with mAbs (Ramarli et al, 1998). NF-κB binding activity was measured by gel shift

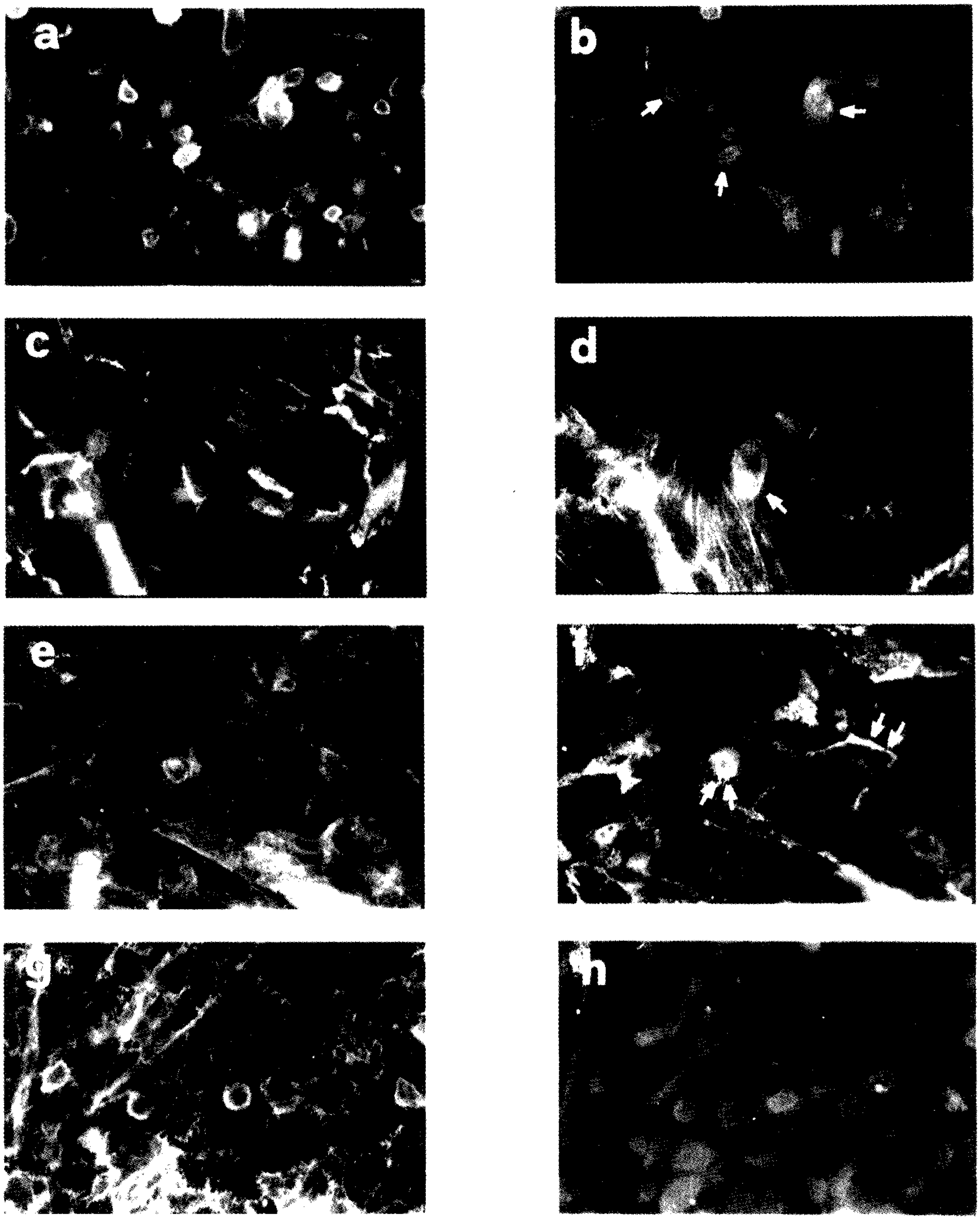

FIGURE 1 Immunostaining of TEC/T cell cocultures Immunostaining of TEC/T cell cocultures for F-actin (a, c,e,g), α4 (b) β1 (d) and α3 integrins (d). α4, β1 and α3 stainings are shown coupled to their F-actin stainings (a, c and e respectively). Frames b, d and f were all focused above the basal surface of TEC monolayers to show the aggregation of β1 (d) and α3 (f) at TEC/T cell interface. Arro heads in (f) indicate show a TEC intercellular boundary that is still in focus. Panels g and h show TEC/T cell cocultures in focus, respectively stained with F-actin (g) and Fitc-labelled 2nd antibody

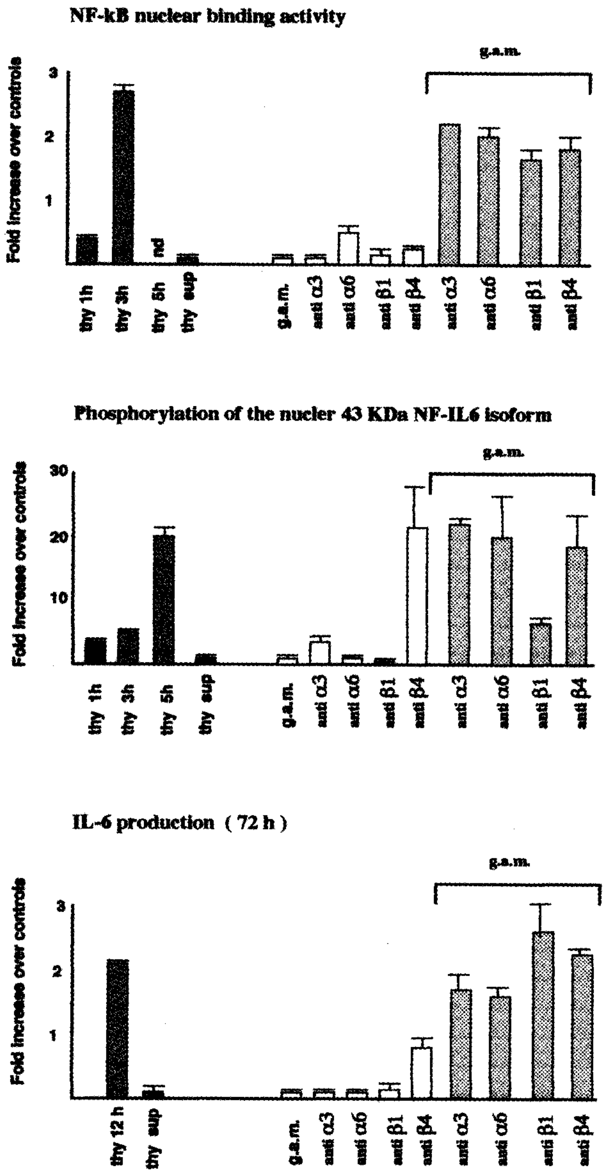

FIGURE 2 Activation of IL-6 transcription factors and IL-6 production in TEC following adhesion with thymocytes or cross-linking of $\alpha3\beta1$ or $\alpha6\beta4$ integrins. Upper panel : NF-KB binding activity of nuclear extracts (6 μg) of TEC untreated, cocultured with thymocyte (thy) for the indicated times, incubated with thymocyte supernatant (thy sup) for 3 h or treated with mAbs anti α (α3,α6) or β (β1,β4) integrins with or without further crossinking with goat anti mouse (g.a.m.). DNA binding activity was quantitated by gel densitometry and expressed as fold increase over controls. Shown are mean values ±SE of results obtained in three different experiments. Middle panel: PAGE analysis of NF-IL6-immunoprecipitates prepared from nuclear extracts of TEC, probed in Western blot with anti phosphoserine mAbs and revealed by ECL. TEC were treated as in the upper panel. Signals of the 36 and 43 KDa bands was quantitated by gel densitometry and expressed as fold increase over controls. Shown are mean values±SE relative to the 43 KDa isoform obtained in three different experiments. Lower panel : IL-6 production by TEC untreated, cocultured for 12 h with thymocytes or incubated with the various mAbs as before. Cross-linking, when applyed, lasted 12 h. OD values were plotted on the internal standard courve, expressed as ng/ml, calculated as ng/$10^6$ cells recovered. Shown are the mean values±SE of the fold increase over controls found at 72 h in two different experiments. IL-6 production by untreated TEC was in the range of 2–20 ngs/$10^6$ cells/day analysis of nuclear extracts obtained from control or stimulated TEC probed with the double stranded oligoprobe containing the IL-6 κB site. Serine phosphorylation of NF-IL6 isoforms was assessed in similar extracts by Western blot analysis of NF-IL6-immune-precipitates probed with anti phosphoserine mAbs and revealed by enhanced chemiluminescence. Shown in the figure are the the fold increase over controls determined by film densitometry. As shown in the upper panel, the adhesion of thymocytes, but not their soluble factors, almost triplicated the NF-κB nuclear binding activity found in the untreated TEC. Similar activity was exerted by the mAb-mediated cross-linking of α (α3, α6) or β (β1, β4) integrins, whereas non cross-linked mAbs failed to function. NF-κB nuclear complexes contained transcriptionally active p50/p65 NF-κB heterodimers (Baldwin AS, 1996), as assessed by the band supershifting obtained in the presence of antisera specifically recognizing p50 or p65 subunits ( not shown). Adhesion of thymocytes or integrin cross-linking (middle panel) greatly increased also the extent of serine-phosphorylation of the NF-IL6 43 and 36 Kda isoforms (the latter not shown) endowed with transactivating activity (Akira S and Kishimoto T, 1997). In contrast to what observed for NF-κB activity, non-crosslinked mAbs anti β4 mAbs were partially effective. The analysis of IL-6 produced by control and stimulated TEC (lower panel) demonstrated that activation of both NF-κB and NF-IL6 transcription was associated with augmented IL-6 secretion, thus indicating that the two phenomena were causally related. It has been demon strated by transfection studies in murine carcinoma cell lines that overexpression of NF-IL6 and the p65 subunit of NF-κB synergistically activates an IL-6 promoter-reporter construct, indicating that these two factors are sufficient to sustain the activation of IL-6 gene (Matsusaka et al, 1993). It has been also demonstrated that p65 subunits is required for maximal gene expression (ibid). We could not assess the relative transactivation of NF-κB and NF-IL6 transcription factors, because TEC stimulated by thymocyte adhesion or cross-linking of α3, α6 and β1 integrins activate the two transcription factors at the same time. Experiments performed with anti β4 mAbs allowed a least to evaluate the NF-IL6 transactivation with or without the cooperation of NF-κB. Results shown in the IL-6 panel indicate that the transactivation induced by NF-IL6 alone was doubled by the presence of NF-κB transcription, thus confirming in normal cells regulated by endogenous transcription factors what previously observed in tumor cells transfected with plasmid constructs. Based on this observation, the T cell inducing activity on TEC IL-6 gene transcription factors was restricted to the time course analysis of NF-κB nuclear binding activity.

### T CELL ADHESION INDUCES NF-κB NUCLEAR BINDING ACTIVITY AND IL-6 PRODUCTION IN TEC

NF-κB binding activity was evaluated in TEC cocultured for 3 h and 12 h with T cells at 1:2 TEC/T cell ratio or treated for 12 h with T cell supernatants. After the removal of T cells (see the Materials and Methods section) TEC were detached, scored negative for $CD2^+$contaminants and used as source of nuclear extracts. TEC aliquots from the 12 h-cocultures were also replated and examined 24 h later. IL-6 secretion was measured in TEC cocultured for 12 h and treated as above. Supernatant from re-plated TEC were collected 24 h and 72 h from the removal of the stimulus. T cell supernatant were prepared from T cell cultured 12 h in TEC medium and used at concentration comparable to the number of cocultured cells. As shown in table III the constitutive NF-κB binding activity of TEC was quickly up-regulated by the T cell contact (2.1±0.4 at 3 h), sustained during the contact (2.3±0.2) and maintained at least up to 24 hrs after the removal of the cell stimulus (1.9±0.3). According to this, the IL-6 production by stimulated TEC was increased 24 h after the removal of the simulus (1.79± 0.7) and still up-regulated 48 h later, as demonstrated by the accumulation observed at the 72 h time point (2.7±1.7). T cell supernatants exerted a negligeable activity ranging 0.01–0.03 fold increase at the two time points.

TABLE III NF-kB activity ([a]) and IL-6 secretion ([a]) in TEC following adhesion of unstimulated T cells

| | *3h* | *12h* | *24h* | *72h* |
|---|---|---|---|---|
| NF-κB nuclear binding activity | 2.1±0.4 | 2.3±0.2 | 1.9±0.3 | nd |
| IL-6 production | nd | nd | 1.79±0.7 | 2.7±1.7 |

a. fold increase ± SE over controls, observed in two different experiments performed with TEC and T cells obtained from different donors. Values were calculated as in Fig. 1 (upper and lower panels)

Taken toghether the presents results demonstrate that mature T cells efficiently reproduce the thymocyte activities as regards their binding to TEC, the involvement of β1 at the membrane level, the induction of clustering of α3β1 integrins at the intercellular boundaries and the up-regulation of IL-6 gene expression associated with NF-κB activation.

### DISCUSSION

Various procedures are currently in use to derive TEC cultures in vitro. Differences concern both the initial steps of the cell isolation from thymic tissues (i.e. enzymatic digestion or explant tecnique) and the expansion in culture (i.e. feeder-layer cell support, serum addition, qualitative composition of complements in the growth medium). All these difference may ultimately result in the preferential expansion of discrete TEC subsets or in culture-induced modifications of expanded cells, hence possibly accounting for the conflicting results often reported in the litterature on TEC expression of surface molecules (i.e. MHC-II antigens, ICAM-1 and VCAM-1) or functional properties ( susceptibility to viral infections).

By using the procedure described in the Materials and Methods section we reproducibly obtained the development of TEC monolayers that display an homogeneous morphology, produce extracellular matrix components (laminins and fibronectin) and maintain unaltered both surface phenotype and morphology up to the $6^{th}$-$7^{th}$ passage of culture, thereafter assuming the spindle-shaped morphology proposed as a marker of late differentiation. The finding that these TEC lack VCAM-1, recently proposed as a marker of cortical cells "in vivo" (Salomon et al, 1998) whereas they uniformly expressed an α3 integrin detected "in vivo" in the medullary regions (Giunta et al, 1991) is suggestive for a medullary rather than a cortical origin. According to reports by others (Boyd et al, 1993) TEC maintained in culture the constitutive production of IL-6 and IL-8 toghether with consistent amounts of Rantes (Ramarli D, personal unpublished observation). It has been reported that cytokines and growth factors induce the IL-6 production by TEC through mechanisms most likely affecting the mRNA stability (Schluns et al 1997).

The first point raised by our work is that IL-6 production can be also up-regulated in TEC at a transcriptional level, by signals delivered by the cross-linking of β1 and β4 integrins at the surface. Integrins are large family of α/β transmenbrane heterodimers able to recognize both extracellular matrix- or cellular ligands and consequently to activate in mesenchimal or lymphoid cells intracellular signalling pathways leading to the expression of genes coding for cytokines or chemokines (IL-1β, IL-6, IL-8, IFNγ) (Defilippi et al, 1997, Mainiero et al, 1998). Signal transduction by integrins has been a matter of intense investigations in the last few years. Reports to date evidenced that clustering of β1 or β4 integrins at the cell surface determines elevation of intracellular pH and $Ca^{2+}$ transients, activation of tyrosinkinases and protein kinase C isoforms, regulation of the Ras and Rho families of the small GTP-proteins and activation of mitogen-activated-protein (MAP) and extracellular-signal-regulated (ERK) kinases ( Defilippi et al, 1997, Giancotti F.G., 1997 ). Less is known on the integrin-mediated activation of gene transcription factors, particularly in normal cells. We have investigated β1 and β4 integrins function in a normal epithelial cell system following ligation and clustering performed with mAbs, treatments which can mimick the integrin engagement with natural ligands.

We demonstrated that the cross-linking of α (α3, α6) or β (β1, β4) chains triggers intracellular cascades able to activate both NF-κB and NF-IL6 transcription factors which, in turn, fulfill the requirements for a maximal transactivation of the IL-6 gene resulting in augmented protein production. Several consideration can be made regarding these results. The first concern the activity of IL-6 produced by TEC, which may of particular relevance within the thymic microenviroment in the light of fact that TEC and thymocytes share the expression of IL-6 receptors. Because of that, integrin regulated or upregulated production of IL-6 may exert dual functions within thymus: on one hand on the survival and/or the cytotoxic differentiation of thymocytes and on the other hand on the differentiation of epithelial cells.

The second concern the role which could be played in TEC by the activated NF-κB and NF-IL6 transcription factors beside the transactivaton of the IL-6 gene. Both transcription factors appear implicated in the control of the survival and/or differentiation of normal and neoplastic epithelial tissues. NF-κB plays a crucial role in the protection from apoptosis in several epithelial and mesenchimal cells (Beg A and Baltimore D, 1996) and, more specifically, in the rescue of rat endothelial cells observed after cross-linking of αvβ3 integrins (Scatena et al 1998). NF-IL6 appears to regulate the proliferation of normal murine hepatocytes (Diehl et al 1998) and the differentiation of normal human keratinocytes and mammary secretory epithelial cells (OH and Smart 1998, Robinson et al 1998). Based on these reports, the finding that β1 and β4 cross-linking activated the two transcription factors suggest that, likewise other epithelial cells types, the TEC adhesion to ECM components may regulate their fate and differention. Within this frame, we have observed in normal TEC that the NF-kB activation induced by the cross-linking of α3β1 integrins partially inhibits TEC from apoptosis following growth factors deprivation (Scupoli et al, 2000).

The third consideration concern the molecular mechanisms underlying the constitutive activation of IL-6 gene transcription factors and the basal production of IL-6 detectable in non stimulated cells. TEC grow in culture forming continous monolayers whose organization is maintained throught the activity of the adhesion receptors that interact at the cell-cell and cell-pastic interface. As demonstrated by immunohystochemistry a3β1 and a6β4 integrins selectively polarize at one or the other location, most likely engaged by ECM proteins and yet unknown cellular ligands. Whether the integrin pools are composed of recycling or stably recruited molecules still need to be elucidated, however, it is conceivable that asyncronous signals delivered by integrins during their ligand recognition may contribute to maintain a basal level of IL-6 production.

The second point raised by our work is that the constitutive production of IL-6 by TEC was strongly up-regulated by the contact with thymocytes or mature T cells which, at the same time, induced the clustering at the TEC contact sites of α3β1 and α6β4 integrins. The lack of detection of ECM proteins at the TEC/thymocyte or TEC/T cell interfaces toghether with the different ability of mAs anti β1 and anti β4 to inhibit the various T cell populations, strongly support the hypothesis that α3β1 and α6β4 integrins espressed by TEC recognize their ligands on the membrane of thymocyte or T cells. The identity of these ligands is at the present unknown. However, binding inhibition studies performed by mAbs indicate that they are differently distributed and/or regulated during the T cell differentiation and activation. Putative ligand/s recognized by α6β4 at the thymocyte surface seem to be restricted to early stages of T cells differentiation or to a microenvironment-dependent expression because they disappeared on mayority of mature T cells. By contrasts, those recognized by α3β1 heterodimers seem shared by thymocytes and mature T cells, thus suggesting a microenvironment-independent, later expression within thymus. Noteworthy, T cell activation negatively influenced this expression. It has been previously shown in keratinocytes that α3β1 integrins can interact homotypically (Symington et al, 1993). This is unlikely to occur at the TEC/T cell interface, because the anti β1 mAbs inhibited the binding of unstimulated T cells, that expressed faint amounts of β1 integrins, but failed in the case of activated T cells which express large amounts of the molecule. Whatever the nature of the cellular ligands, they efficiently recruit integrins able to activate in TEC the signalling pathways leading to activation of NF-κB and NF-IL6 and IL-6 gene expression thereby implying that this recognition is one of the mechanisms underlying the inducing activity of immature or mature T cells. It has been demonstrated that ECM adhesion and de-adhesion regulate the cell positioning within tissues, their survival or differentiation. We propose here that heterotypic cell-cell adhesion thank to the activity of the same adhesion receptors may cooperate in or finely tune the same processes.

### *Acknowledgements*

We gratefully aknowledge Dr. Marcello Merola for helpful discussion and suggestions concerning transcription factors studies and the Cardiosurgery Division at Az. Ospedale Verona-University of Verona for providing thymic specimens. This work was partially supported bt ISS-AIDS No. 9306.34 (1995) to GT and by 1178 Telethon grant to DR.

The laboratory of PCM was supported by AIRC, MURST and Telethon grants.

### *References*

Adkins B., Chun K., Hamilton K., and Nassiri M. (1996) Naive murine neonatal T cells undergo apoptosis in response to primary stimulation. J.Immunol. 157 : 1343–1349.

Akira S, and Kishimoto T. (1997). NF-IL6 and NF-kB in cytokine gene regulation. Adv in Immunol 65: 46.

Baldwin A.S. (1996) The NF-kB and IkB proteins : new discoveries and insights. Annu. Rev. Immunol. 14: 649–681.

Beg A and Baltimore D. (1996) An essential role for NF-kB in preventing TNF-α-induced cell death.Science 274 : 782–784.

Branderberger R., Kammerer R.A, Engel J., and Criquet M. (1996). Native chick laminin-4 containing the β2 chain (s-laminin) promotes motor axon growth. J. Cell Biol. 135: 1583–1592.

Delwel G.O., de Melkerl A.A., Hogervorst F., Jaspals L.H., Fles D.L., Kuikman I., Lindblom A., Paulsson M., Timpl R., and Sonnemberg A. (1994) Distinct and overlapping ligand specificities of the α3β1 and α6β1 integrins: recognition of laminin isoforms. Mol. Biol. Cell 5: 203–215.

Boyd R.L., Tucek C.L., Godfrey D.I., Izon D.J., Wilson T.J., Davidson N.J., Beam A.G.D., Ladyman H.M., Ritter M.A. and Hugo P. (1993). The thymic microenvironment. Immunol Today 14: 445–459.

Cohen-Kaminski S., Devergne O., Delattre R.M., Klingel-Schmitt I., Emilie D., Galanaud P., and Berrih-Aknin S. (1993). Interleukin 6 overproduction by cultured thymic epithelial cells from patients with myasthenia Gravis is potentially involved in thymic iperplasia. Eur Cytokine Net. 4: 121–132.

Couture C., Amarante-Mendes G., and Potworowsky E. (1992). Tyrosin kinase activation in thymic epithelial cells : necessity of thymocyte contact through the gp23/45/90 adhesion complex. Eur J Immunol 22: 2579–2585.

Defilippi P., Gismondi A., Santoni A., and Tarone G. (1997) Signal Transduction By Integrins (Landes Bioscience Springer Publishing Company Austin, TX).

Diehl AM. (1998) Roles of CCAAT/enhancer-binding proteins in regulation of liver regenerative growth J biol Chem 273: 30843–30846.

Giancotti F.G. (1997) Integrin signaling:specificity and control of cell survival and cell-cycle progression. Curr Opin Cell Biol 9: 691–700.

Giunta M., Favre A., Ramarli D., Grossi C., and Corte G. (1991) A novel integrin is involved in thymocyte-thymic epithelial cell interaction. J Exp Med 173: 1537–1548.

Green H., Kehinde O., and Thomas J. (1979). Growth of cultured human epidermal cells into multiple epithelia suitable for grafting. Proc Natl Acad Sci USA 76: 5665–5670.

Hadden JW. (1998). Thymic endocrinology. Ann N Y Acad Sci 840: 352–358.

Henttinen T., Levy D.E., Silvennoinen O., and Hurme M. (1995) Activation of the signal trasducer and transcription (STAT) signaling pathway in a primary T cell response. J. Immunol. 155: 4582–4587.

Hollander G.A., Wang B., Nichorgiannopoulou A., Platemburg P.P., van Ewijk W., Burakoff S.J., Gutierrez-Ramos J.C. and Terhorst C. (1995). Developmental control point in induction of thymic cortex regulated by a subpopulation of prothymocytes. Nature 373: 350–353.

Lee M.G., Sharrow S.O., Farr A.G., Singer S., and Udey M.C. (1994). Expression of the homotypic adhesion molecule E-cadherin by immature murine thymocytes and thymic epithelial cells. J.Imunol 152 : 5653–5659.

Mainiero F., Gismondi A., Soriani A., Cippitelli M., Palmieri G., Jacobelli K., Piccolo M., Frati L., and Santoni A. (1998) Integrin-mediated Ras-extracellular regulated kinase (ERK) signaling regulates interferon-γ production in human natural killer cells. J exp Med 188: 1276–1275.

Matsusaka T., Fujikawa K., Nishio T., Mukaida N., Matsushima K., Kishimoto T., and Akira S. (1993) Transcription factors NF-IL6 and NF-κB sinergistically activate transcription of the inflammatory cytokines, interleukin 6 and interleuchin 8. Proc Natl Acad Sci USA 90: 10193–10198.

OH H.S. and Smart R.C. (1998) C/EBPβ is associated with squamous differentiation in epidermis and isolated primary keratynocytes and is altered in skin neoplasms. J Invest Dermatol 110: 939–945.

Patel D.D. and Haynes B.F. (1993). Cell adhesion molecules involved in intrathymic T cell development. Semin Immunol 5: 283–292.

Penit C., Lucas B., Rieker T., and Boyd R.L. (1996). Thymic medulla acquire specific markers by post-mitotic maturation. Dev Immunol 5: 25–36.

Ramarli D., Reina S., Merola M., Scupoli M.T., Poffe O., Riviera A.P., Brentegani M., Fiorini E., Vella A., Varnier O., and Tridente G. (1996). HTLV-IIIB Infection of human thymic epithelial cells: viral replication correlates with the induction of NF-κB binding activity in cells activated by cell adhesion. AIDS Res Hum Retrovir 12: 1217–1225.

Ramarli D., Scupoli M.T., Fiorini E., Poffe O., Brentegani M., Villa A., Cecchini G., Tridente G., and Marchisio P.C. (1998) Thymocyte contact or mAb-mediated clustering of α3β1 or α6β4 integrins activate IL-6 transcription factors (NF-κB and NF-IL6) and IL-6 production in human thymic epithelial cells. Blood 92: 3745–3755.

Robinson G.W., Johnson P.F., Hennighausen L., and Sterneck E. (1998) The C/EBP β transcription factor regulates epithelial cell proliferation and differentiation in the mammary gland. Genes Dev 12 : 1907–1916.

Savino W., Serra Villa-Verde D.M., Alves L.A., and Dardenne M. (1998). Neuroendocrine control of the thymus. Ann N Y Acad Sci 840: 470–479.

Salomon D., Crisa L., Mojcik C.F., Ishii J.K., Klier G., and Shevach E.M. (1997). Vascular adhesion molecule-1 is expressed by cortical thymic epithelial cells and mediate thymocyte adhesion. Implications for the function of α4β1 (VLA4) integrin in T-cell development. Blood 89: 2461–2471.

Scatena M., Almeida M., Chaisson M.L., Fausto N., Nicosia R.F. and Giachelli C.M. (1998). NF-κB mediates αvβ3 integrin-induced endothelial cell survival. J Biol Chem 141: 1083–1093.

Screpanti I., Meco D., Scarpa S., Morrone S., Frati L., Gulino A., and Modesti A. (1992). Neuromodulatory loop mediated by nerve growth factor and interleukin 6 in thymic stromal cell cultures. Proc Acad Sci USA 89: 3209–3212.

Screpanti I., Scarpa S., Meco D., Bellavia D., Stuppia L., Frati L., Modesti A., and Gulino A. (1995). Epidermal growth factor promotes a neural phenotype in thymic epithelial cells and enhances neuropoietic cytokine expression. J Cell Biol 130 : 183–192.

Schluns K.S., Cook J.E., and Le P.T. (1997) TGF-beta differentially modulates epidermal growth factor-mediated increases in leukemia-inhibitory factor, IL-6, IL-1 alpha, and IL-1 beta in human thymic epithelial cells. J Immunol 158: 2704–2712.

Schreiber E., Matthias P., Muller M.M., W. Schaffner W. (1989). Rapid detection of octamer binding proteins with "mini extracts" prepared from a small number of cells. Nucleic Acids Res 17: 6419.

Sarpoli H.T., Fiorini E., Marchisio P.C., Poffe O., Tagliabue E., Brentegani M., Tridente G., Ramarli D. (2000). Lymphoid adhesion promotes human thymic epithelial cell survival via NF-xB activation. J. Cell Science 113 : 169-177.

Symington B.E., Takada Y., and Carter W.C. (1993). Interaction of integrins α3β1 and α2β1: potential role in keratinocyte intercellular adhesion. J Cell Biol. 120: 523–535.

